# An Unusual Case of Pyrexia of Unknown Origin

**DOI:** 10.7759/cureus.16684

**Published:** 2021-07-28

**Authors:** Mina Soliman, Ahmad Shirazi-Nejad, Dominic Bullas, Elmuhtady Said

**Affiliations:** 1 Medicine, NHS Hospital, London, GBR; 2 Gastroenterology, Barnsley Hospital NHS Foundation Trust, Barnsley, GBR

**Keywords:** crohn’s disease (cd), puo, lower abdominal pain, fever with rash, non-bloody diarrhea

## Abstract

A previously fit and well 29-year-old man with no significant recent travel or contact history presented to the hospital with 11 days of feeling unwell, intermittent diarrhea, abdominal pain and a skin rash that was consistent with folliculitis. Despite resolution of these index symptoms he continued with recurring fever of 38.5 degrees centigrade and weight loss of six Kilograms over the next three weeks. Extensive investigations to find a cause for the unexplained persistent fever failed to reveal an etiology, hence fulfilling pyrexia of unknown origin definition (PUO). None of the three main causes of PUO, namely infections, autoimmune diseases or underlying malignancy, were confidently found. Colonoscopy was suggested following a review of the abdominal CT scan to investigate possible thickening of the bowel wall. A diagnosis of atypically presenting Crohn’s disease was eventually made and confirmed by colonoscopy and histology. The fever responded promptly to treatment of the Crohn's disease and he remained well at follow-up at six and 12 months after the initial presentation. In conclusion, it is important to keep in mind that PUO can be a rare initial presentation of inflammatory bowel disease in young adults with little or no gastrointestinal symptoms.

## Introduction

Crohn’s disease (CD) is a chronic inflammatory bowel disease (IBD) that can involve any part of the gastrointestinal tract [1]. The annual incidence of CD in the UK is increasing especially among adolescents, with a reported prevalence of 397/100,000 people [2]. The usual age of presentation is in late teens to young adults. Crohn's colitis often presents with diarrhea, which can be bloody. Weight loss can be a feature associated with the diarrhea. It can also present with small bowel obstructive type symptoms, i.e. sickness, vomiting and abdominal pain. Perianal involvement can lead to fistulae and/or abscess formation. Extra-intestinal manifestations affect the eye, skin or joints and occur in up to 31% of patients [3]. Crohn's disease is usually classified according to the Montreal Classification. The three main characteristics are age at onset, site of disease and disease behavior, with perianal involvement being a further subset [4]. Pathologically, Crohn's can be non-stricturing, non-penetrating; stricturing; or a third type which is penetrating [4]. Fever is an uncommon feature in Crohn’s disease, which if present is usually associated with gastrointestinal symptoms.

## Case presentation

A 29-year-old factory worker was admitted to the hospital following a referral from his General Practitioner. He had been unwell for 11 days, experiencing symptoms of lethargy, fever, skin rash, lower abdominal pain and intermittent diarrhea. There was no vomiting or rectal bleeding. No risk factors for food poisoning were elucidated. Also, there was no history of recent foreign travel or contact with people who were ill. His past medical history included well-controlled asthma and he was otherwise fit and well. He was a non-smoker and rarely drank alcohol. His family history was unremarkable. Examination on admission revealed: tachycardia of 112 beats per minute, respiratory rate of 17 breaths per minute, temperature of 38°C, blood pressure 149/102 mm Hg. Oxygen saturation and capillary refill time were within normal limits. Clinical assessment revealed unremarkable cardio-respiratory examinations. Skin examination showed generalised pustular rash on the limbs and trunk. Abdominal examination revealed a soft non-tender abdomen.

Blood tests showed raised white cell count at 16.2 x109/L, neutrophil count of 11.4 x109/L, haemoglobin (Hb) 128g/L and normal platelet count, renal function and liver enzymes. Inflammatory markers were elevated with C-reactive protein (CRP) 120mg/L and erythrocyte sedimentation rate (ESR) 99mm/hour. Infection screen revealed negative urine microscopy, stool and blood cultures. Virology screen was also negative including HIV and Epstein-Barr virus (EBV) serology. Autoimmune screen including autoantibody, double strand DNA, complement and immunoglobulin came back within normal. Chest and abdominal X-rays were unremarkable. Due to the fever and the folliculitis he was initiated on intravenous co-amoxiclav 1.2 grams three times per day. The rash improved significantly after three days of his admission and the diarrhea settled completely but the fever persisted. Bowels were opening two to three times per day with type 4 stools on the Bristol chart. Faecal calprotectin was not done due bowel being back to complete normality.

Due to persistence of fever, CT scan of the abdomen and pelvis was done and excluded intra-abdominal abscesses, collections or discitis. However, it did reveal numerous prominent intra-abdominal lymph nodes, the largest (17mm) overlying the anterior margin of the right psoas muscle. The rest of the examination was unremarkable; in particular there was no mention of pathology of the large or the small bowel. Echocardiogram revealed no evidence of valvular vegetations or infective endocarditis. His pro-calcitonin was normal at 0.21 ng/ml, consistent with non-infective cause for the fever. In view of the lymphadenopathy on the CT scan, Haematology and Rheumatology input were sought. Their conclusion was that there was no evidence to suspect either lymphoma or an underlying systemic collagen disorder.

**Figure 1:**
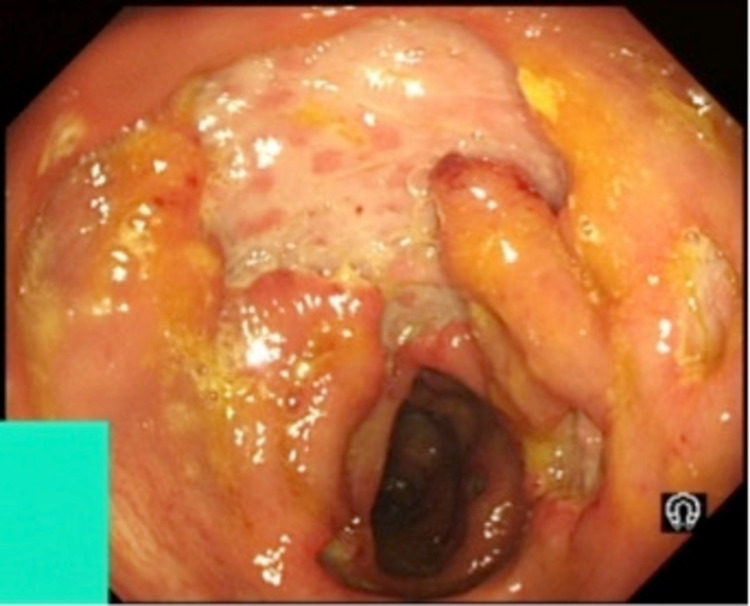
Colonoscopy: distal descending colon showing deep ulceration.

Throughout this period of investigation, he remained pyrexial with daily increase in temperature to 38.5 C and experienced 6-kilogram (kg) weight loss. Dietitian review added nutritional supplements in the form of regular ensure. After three weeks, his fever fit the criteria for pyrexia of unknown origin definition (PUO) [5]. The CT images were re-visited in the X-ray meeting. The review queried whether the caecum looked slightly thickened. The enlarged lymph nodes appeared reactive. The decision was therefore made to progress to colonoscopy to inspect the caecum and also to do MRI small bowel. The MRI of the small bowel was normal. Colonoscopy was done three weeks after his admission and almost five weeks after symptoms onset. This showed multiple, patchy, punched-out ulcers affecting the sigmoid and descending colon; the rest of the large bowel showed some patchy inflammation. Terminal ileum was normal (Figure 1).

The appearance raised the possibility of Crohn’s colitis or cytomegalovirus (CMV) infection. The endoscopic appearance in combination with folliculitis also raised the possibility of intestinal Behçet’s, but this was excluded after histological review of the biopsies and absence of other features of Behçet. CMV IgG was positive but CMV IgM and viral DNA titre were negative. Colonic biopsies showed chronic active inflammation with cryptitis and crypt abscess with plasma cells predominance affecting the full thickness in some of the biopsies. The biopsy appearance was consistent with Crohn’s disease.

After positively diagnosing Crohn's colitis, the patient was commenced on IV hydrocortisone 100mg four times a day and made a significant improvement and became apyrexial. In view of the deep ulceration and the aggressive phenotype, the IBD MDT decision was to follow a step down approach and to start infliximab (anti-TNF) treatment, after counselling the patient, to achieve and maintain full remission. He was discharged home almost four weeks from his admission. At follow-up six weeks post-discharge, his blood parameters were normalised and he had gained weight. He remained asymptomatic at six and 12 months.

## Discussion

PUO is defined as persistent fever lasting three weeks or more, without obvious cause, despite thorough investigations. The three main causes of PUO are infections, malignancy and autoimmune diseases. Even though fever and systemic upset can be present during an inflammatory bowel disease (IBD) flare-up, it is usually associated with other features, such as diarrhea and passage of blood per rectum. Prolonged fever is unusual even with typical symptoms of inflammatory bowel disease, as is presented in a case report by Fei Zhou et al, in which the patient had diarrhea for three months and fever/night sweats for two months [6]. A new diagnosis of IBD presenting as PUO is very rare. Only a few case reports in the literature link IBD and PUO together, however these were mainly in paediatric [7] or elderly populations. Shpilberg presented a case of a 71-year-old gentleman with PUO, who was diagnosed as ulcerative colitis (UC) following a PET scan showing significant uptake in the left colon [8]. This report advises that in the elderly population, ulcerative colitis (UC) should be considered as a cause of PUO, even in the absence of gastrointestinal symptoms. In adult populations, Muñoz et al. (2007) reported two patients with Crohn's disease who presented with PUO, however both had mild intestinal symptoms and one had polyarthritis [9]. Our case had only short-lived diarrhea and abdominal pain at the onset, but a paucity of gastrointestinal symptoms for the majority of remaining time.

It is not clear whether the folliculitis was also part of the unusual Crohn’s presentation. However, it did clear within one week of hospital admission while the pyrexia persisted for a further three weeks until Crohn’s disease was diagnosed and treated. Furthermore, it is not a skin condition that is known to be associated with IBD, given that the main cutaneous extra-intestinal manifestations of IBD are erythema nodosum, pyoderma gangernosum, Sweet syndrome and aphthous stomatitis [10].

Finally, some infections, viral or bacterial, can present with a skin rash and PUO. These were excluded in our case either by lack of relevant travel history or by serology. Adult-onset Still's disease is one condition that can also present with skin rash and PUO, however the rash is usually macular or maculopapaular and not folliculitis type. In addition, this condition is usually associated with musculoskeletal symptoms in the form of arthralgia and/or arthritis [11].

## Conclusions

Pyrexia of unknown origin usually imposes a significant diagnostic challenge due to the large number of possible causes. A thorough history and clinical examination along with awareness of uncommon association is important to target investigations. PUO can be a rare initial presentation of inflammatory bowel disease. One should be also mindful that in such cases, inflammatory bowel disease can present with sparse or absent gastrointestinal tract symptoms.
